# Consortium‐Based Patient and Public Involvement and Engagement for Long COVID Research: A Pirit‐Focused Impact Evaluation of the PHOSP‐COVID Study

**DOI:** 10.1111/hex.70591

**Published:** 2026-03-20

**Authors:** Linzy Houchen‐Wolloff, Joanna Bell, Rebecca Pritchard, Krisnah Poinasamy, Kate Holmes, Samantha Walker, Nikki Smith, Claire Hastie, Natalie Rogers, Dawn Adams, Rashmita Nathu, Rhyan Gill, Jenny Bunker, Lily Staunton, James Chalmers, Ling‐Pei Ho, Victoria Harris, Alexander Horsley, Michael Marks, Betty Raman, Louise V. Wain, Christopher Brightling, Rachael Evans

**Affiliations:** ^1^ NIHR Leicester Biomedical Research Centre Leicester UK; ^2^ Asthma and Lung UK London UK; ^3^ National Institute for Health Research London UK; ^4^ PHOSP‐COVID Patient and Public Involvement Group, Leicester Biomedical Research Centre Leicester UK; ^5^ Long Covid Support London UK; ^6^ University of Dundee, Ninewells Hospital and Medical School Dundee UK; ^7^ MRC Human Immunology Unit, University of Oxford Oxford UK; ^8^ Division of Infection, Immunity & Respiratory Medicine, Faculty of Biology, Medicine and Health University of Manchester Manchester UK; ^9^ Department of Clinical Research London School of Hygiene & Tropical Medicine London UK; ^10^ Radcliffe Department of Medicine University of Oxford Oxford UK

**Keywords:** engagement, impact, long COVID, patient involvement

## Abstract

**Background:**

At the start of the coronavirus disease‐2019 (COVID‐19) pandemic in early 2020, the long‐term outcomes for survivors of COVID‐19 were unknown. The PHOSP‐COVID cohort study was set up at scale and pace in Spring 2020 to determine the short‐ to long‐term health consequences of COVID‐19 in post‐hospitalisation survivors; to understand the impact of interventions during and after the acute illness on these long‐term sequelae and to build the foundation for multiple in‐depth studies. A consortium infrastructure of hospital trusts, academic partners, industry, patients and charities was created. From the study inception, patients were central to the PHOSP‐COVID consortium, whereby a Patient and Public involvement and Engagement (PPIE) group was convened, including charity groups, people with lived experience recruited through clinical care from NHS sites, and patient support groups through Long Covid Support. Embedding high‐quality, meaningful PPIE within a large consortium brings challenges and benefits. In this article, we describe our experiences of setting up and sustaining the PHOSP‐COVID Consortium PPIE group, including a PIRIT‐focussed evaluation of the impact of our PPIE work and provide top tips for researchers to take forward when embedding PPIE in future consortium research.

**Methods:**

This article outlines the set‐up and sustainability of the PHOSP‐COVID study PPIE group, in consultation with the National Institute for Health and Care Research (NIHR) guidance. To evaluate PPIE impact, we used PIRIT (Public Involvement in Research Impact Tool, 2023‐ Cardiff), and we provide our honest reflections of our PPIE work according to the PIRIT planning tool. The results highlight the benefits of a consortium approach to PPIE as well as the challenges, with quotes from PPIE contributors and academics. In addition, we have created top tips for researchers to take forward when embedding PPIE in future consortium research, linked to the NIHR standards.

**Learning and Reflection:**

This manuscript has identified gaps in PPIE considerations for the PHOSP‐COVID study and specific challenges around a consortium‐based approach for PPIE. These are largely due to time scale (i.e. the pace of setting up the study within a pandemic) and communication factors (diverse and large numbers of people to include/inform). Through reflection on the challenges and successes experienced in the PHOSP‐COVID consortium PPIE via a PIRIT‐focused impact evaluation, we have developed recommendations to support future good practice.

**Patient or Public Contribution:**

Patients and members of the public were involved in all aspects of this work from idea inception, design and conduct of the work, analysis and interpretation of the data. Eight patients prepared the manuscript and are included as co‐authors.

**Abbreviations**
ALUK: Asthma + Lung UK
ARC: Applied Research Collaboration
BRC: Biomedical Research Centre
ISRCTN: International Standard Randomised Controlled Trial Number
NDA: Non‐disclosure agreement
NHS: National Health Service
NIHR: National Institute for Health and care Research
PHOSP‐COVID: Post‐hospitalisation COVID‐19 study
PHOSP‐I: PHOSP Inflammation
PiiAF: Public Involvement Impact Assessment Framework
PIRIT: Public Involvement In Research Impact Toolkit
PPIE: Patient and Public Involvement and Engagement
SARS: Severe Acute Respiratory Syndrome

## 1 Background

The global social movement towards public involvement, founded in democratic principles, seeks to deliver purposeful involvement that impacts research in the direction of: ethical and social acceptability; accessibility; relevance; appropriateness to the public [1]. Patient and Public involvement and Engagement (PPIE) is an active partnership between patients, the public and researchers throughout the research process, which aims to ensure research is relevant, accessible, beneficial to patients and the public, and ultimately contributes to improved healthcare. Patients offer unique insights from their lived experiences, which cannot be substituted by knowledge from clinicians, researchers or other specialist stakeholders.

As SARS‐CoV‐2 was a new challenge, patients’ insights into understanding the consequences of coronavirus disease‐2019 (COVID‐19) and the longer‐term sequelae were critical [2]. Indeed, the first descriptions and naming of Long Covid came from people with lived experience [3].

At the start of the pandemic in early 2020, the long‐term outcomes for survivors of COVID‐19 were unknown, but lessons learned from other pandemics [such as the Severe Acute Respiratory Syndrome (SARS)] indicated that some survivors would have physical and mental health complications that would require follow‐up [4]. The PHOSP‐COVID cohort study was therefore established at scale and pace with three broad aims to:

1. Determine the short‐ to long‐term chronic health (and health economic) sequelae of COVID‐19 infection in post‐hospitalisation survivors; to define demographic, clinical and molecular biomarkers of the susceptibility, development, progression and resolution of these health sequelae.
2. Understand the impact of interventions during the acute illness on these long‐term sequelae.
3. Build the foundation for multiple in‐depth studies e.g. lung fibrosis, pulmonary and systemic vasculature, cardiometabolic, renal, sarcopenia, rehabilitation, mental health and neurological disease.

A consortium infrastructure was established, including National Health Service hospital trusts, academic partners, industry, patients and charities (www.PHOSP.org). From the start, patients were central to the PHOSP‐COVID consortium and a PPIE group was convened, including charity groups, people with lived experience recruited through clinical care from NHS sites, and later through patient support groups, namely Long Covid Support. Commonly, public involvement in research is focused predominantly on specific aspects of individual research studies such as the initial study set‐up, study methods, data analysis, report writing and dissemination. PHOSP‐COVID sought to involve patients and the public not only throughout the project life‐cycle but to achieve integration of the project PPIE into the infrastructure of the consortium.

Barriers or challenges to traditional study PPIE are well documented from patients’ perspectives: poor communication, lack of knowledge of PPIE opportunities, time constraints and lack of support; and from researchers’ perspective: balancing inputs and managing relations, time, and resources and training [5]. There is emerging data to suggest additional challenges to undertaking PPIE in Long Covid [6], particularly around the use of digital tools to facilitate remote working. Whilst for some, digital tools allow greater accessibility for those unable to leave the home, for others digital exclusion can occur if they lack essential skills and devices required to engage with technology. However, there is little information on how to undertake PPIE within a consortium, which has the complexity of communicating with members on a large scale, beyond that of a traditional research study. Furthermore, public involvement impact assessment data remain relatively scarce or inconsistently collected [1], yet there are many tools available to measure PPIE impact.

## 2 Aims

We aimed to first describe our experiences of setting up and sustaining the PHOSP‐COVID Consortium PPIE group, second to provide a PIRIT‐focussed evaluation of impact on the PHOSP‐COVID study PPIE and third to provide our reflections and lessons learned for researchers to take forward when embedding PPIE in future consortiums or pandemic research.

## 3 Methods

### 3.1 Setting up the Study and the PPIE Group

**Figure 1:**
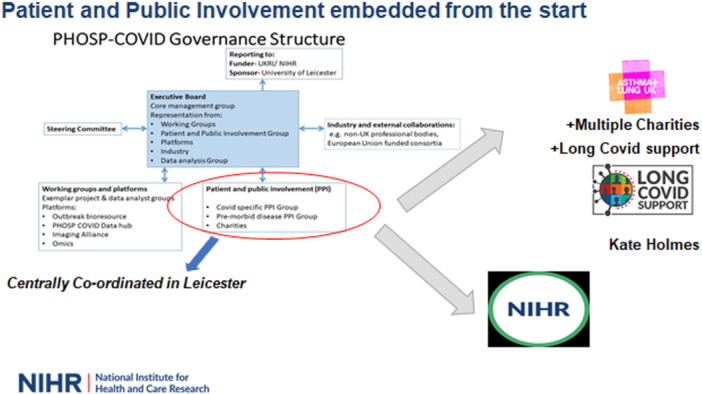
PHOSP‐COVID governance structure with embedded PPIE.

**Figure 2:**
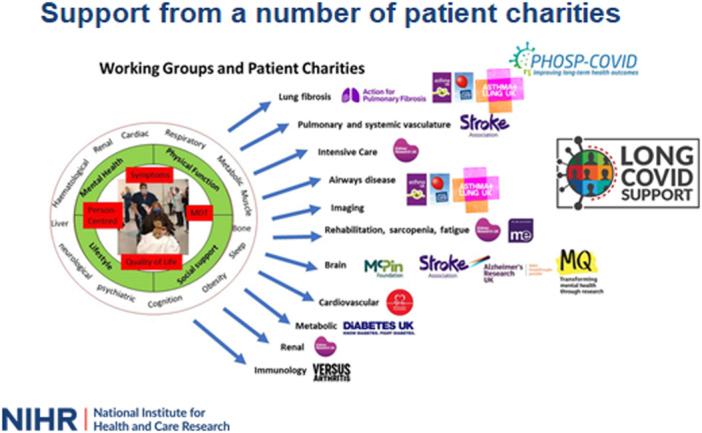
Patient charities’ linkage to PHOSP‐COVID working groups.

The PHOSP‐COVID study is a UK‐wide national research collaboration examining the long‐term sequelae of COVID‐19 (https://www.PHOSP.org). Over 7500 patients discharged from over 60 UK hospitals between March 2020 and March 2021 were recruited and followed up for 12 months (Ethics approval: Leeds West Research Ethics Committee, Reference 20/YH/0225 and Trial Registration: https://doi.org/10.1186/ISRCTN10980107) [7]. The governance structure for the PHOSP‐COVID Consortium is shown in Figure 1 and highlights the foresight that PPIE was embedded from the start of the study. This was enabled by creating a PHOSP‐COVID Charities Group consisting of a diverse range of 13 charities who worked with the PHOSP‐COVID Executive Board and disease‐specific Working Groups to ensure that the patient voice was taken into account at all stages of the study. Charities (see https://phosp.org/charity-society/) were selected to represent specific organ areas and were participating members of the Working Groups where they provided expertise and helped to ensure that research priorities reflected those of their communities (Figure 2). The Charities Group was led by Asthma + Lung UK (ALUK‐ formally The British Lung Foundation and Asthma UK) and the National Institute for Health and Care Research (NIHR). ALUK brought into the consortium Long Covid Support, a national charity that supports more than 67,000 people living with Long Covid. PPIE was coordinated centrally by the Leicester Biomedical Research Centre (BRC), who added the voice of lived experience by inviting those who were recruited to the PHOSP‐COVID study to join the group.

In forming our group, we used the 4 priority areas for PPIE development from the *Going the Extra Mile* document [8] and the 6 NIHR standards [9]. We evaluated how these principles and standards were considered in our work.

### 3.2 Impact Assessment

**Figure 3:**
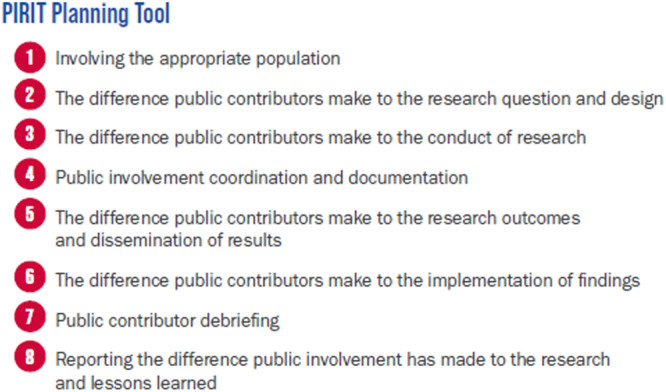
PIRIT planning tool.

A number of tools may be used to evaluate PPIE impact, and we considered the following: GRIPP2 reporting checklist [10], PIRIT [11] and Public Involvement Impact Assessment Framework [12]. PIRIT was selected to assess our PPIE impact and ongoing work (Figure 3) as a result of a joint conversation with our research team and the PPIE contributors. “PIRIT” refers to the Public Involvement in Research Impact Toolkit, a set of free, pragmatic tools designed to help researchers and public contributors plan, track, and evaluate the impact of public involvement in research. The rationale was that this had a simple spreadsheet to record when and how the public contributed, what they hoped to influence, what changed (if anything), why it matters, and allows reporting of impact against the UK Standards for Public Involvement [9]. Using PIRIT for reflection involves dialogue between researchers and public contributors, critical thinking about the experience, and documenting instances where public input led to changes. To do this, we met online as group of researchers and PPIE contributors to consider the individual PIRIT questions. The draft of responses formed the basis of this manuscript and were critically revised by all authors. The full question set that we evaluated can be found at: https://engage.hscni.net/site/wp-content/uploads/2024/12/PIRIT_Planning-Tool_V1_0_FINAL.pdf.

## 4 Results

Results are presented according to honest reflections of our PPIE work according to the PIRIT planning tool by all authors (patients and researchers) and reflect the whole time course of the PHOSP‐COVID study from set‐up, through to legacy work.

### 4.1 Involving the Appropriate Population

**1a. Public contributor opportunities and involvement mechanisms and 1b. Public contributor recruitment**

In the first 6 months, PPIE contributors were selected from the patient charities, to provide a national perspective and from Leicester participants who had taken part in the trial, to provide a local voice. Early consideration of PPIE from the inception of the PHOSP‐COVID consortium and embedded within the governance structure underpinned effective implementation. Consequently, PPIE was integral to the overall management of the study. This was operationalised by identified PPIE leads (co‐authors KP, SW, KH and LHW) being a connector between the PPIE group and the PHOSP‐COVID study Executive team. They reported on PPIE activities, issues and engagement at quarterly Executive meetings.

As the PHOSP‐COVID consortium was brought together quickly, there was no PPIE co‐applicant on the original grant application. The PPIE group initially lacked diversity in terms of protected characteristics, particularly for gender and ethnicity beyond White British individuals. Subsequently, diversity was improved by targeting invites to patients who had taken part in the trial in Leicester rather than our national consortium/charities group. This included increasing the number of males in the group from 0 to 3 and increasing non‐White British individuals from 0 to 2. This proved fruitful as Leicester is one of the most diverse cities in England, with no single ethnic group making up the majority of its population [13]. Furthermore, digital literacy/access may have precluded a number of potential PPIE members from joining the PPIE group. Conversely, digital access aided accessibility for patients managing symptoms and/or other commitments. Membership of the group reduced over time by approximately 20%, highlighting that an ongoing recruitment strategy to bring new members into the PPIE group would have been potentially effective, particularly to avoid overburdening public contributors and to add the views of those developing Long Covid from later Covid‐19 variants. A written PPIE member role description may also have been useful. This was done informally through discussion, but a written document may have been helpful to manage expectations. Our local Applied Research Collaboration (ARC East Midlands) suggests that the level of expectation placed on PPIE partners increases when collaborating, so they encourage researchers to develop a ‘role, responsibilities and expectations’ document for researchers and PPIE partners alike [14]. This wasn't something we formalised into a document for this project, but roles and expectations were discussed.

**1c. Costing and funding public involvement**

Public contributors were reimbursed for their time in terms of the hours spent at a value per hour, in line with the recommended NIHR rate: (https://www.nihr.ac.uk/payment-guidance-researchers-and-professionals). Time was reimbursed for work conducted within and outside of meetings, including preparatory time for meetings and reviewing documents, etc.

Although PPIE participants’ time was reimbursed, this was via bank transfer at set time periods throughout the year, broadly quarterly, in line with our usual NHS trust processes at the time of set‐up. It may have been beneficial, where possible, to ask participants if they would rather have vouchers than money and also their preferred frequency of payment. An itemised summary, sent to payees of what is being reimbursed i.e. a list of meetings and tasks that each specific payment covers, would also be welcomed in future. This would need to be considered in the context of advice from Human Resources, as PPIE payments can have taxation implications.

**1d. Public contributors’ support and training**

Expectations of the group were established early in the process, through discussion of the group's purpose and remit; ensuring participants understood their role, and how their time would be utilised and reimbursed, although, as noted, a written role description was not created. This has been key to creating a mutually beneficial experience for members and researchers. Participants were also trained in PPIE skills e.g. reviewing documents and had access to the Leicester BRC PPIE team for support, though this was rarely accessed.

### 4.2 The Difference That Public Contributors Make to the Research Question and Design

**2a. Informing research priorities and 2b. Shaping the research questions**

The PHOSP‐COVID consortium was in a unique position to establish the top research priorities for survivors of a hospital admission with COVID‐19 [15], with an exercise conducted from December 2020 to March 2021. This used an abridged version of the well‐established James Lind Alliance process (see: https://www.jla.nihr.ac.uk/video/psp-process), due to the time pressures of the PHOSP‐COVID study, and this being a new disease. The PPIE group was key in refining the question list for the online survey and final workshop. Thirteen patients attended the final priority setting workshop, importantly equal in number to the clinicians/researchers in attendance, to ensure a balanced discussion. The final top 10 priorities represented the key questions at the time around identifying underlying mechanisms of Long Covid, establishing diagnostic tools, understanding the trajectory of recovery and evaluating the role of interventions during both the acute and persistent phases of the illness. These priorities were then reported back to the PHOSP‐COVID working groups, who have utilised the priorities to drive their work. Any published papers from the PHOSP‐COVID Consortium referenced (and continue to reference) the priority the work addresses. However, these may have changed since the early pandemic.

**2c. Research methods’ acceptability and accessibility**

Patient contributors were involved in pilot testing study processes (e.g. electronic consent), study documents (e.g. patient information sheets) and data collection support tools (e.g. sputum sample collection video). Changes were made in response to PPIE member feedback.

**2d. Defining the study outcomes and measures**

Due to the pace and scale of set‐up, public contributors were not included in the initial discussions about the selection of study outcome measures. However, once the PPIE group was established, any changes to the study protocol were discussed and agreed upon with input from the members.

### 4.3 The Difference Public Contributors Make to the Conduct of Research

**3a. Ethical considerations and 3b. Study participant recruitment strategy**

As previously stated, we were unable to include public contributors in the planning or ethical approval phases of this study. A tension with our PPIE group was that the PHOSP‐COVID study was specifically funded to only follow up patients who had been hospitalised with their acute COVID‐19 infection. However, a large proportion of people with Long Covid have not been hospitalised. This left some members of the group feeling that the non‐hospitalised cohort had been excluded and left behind in terms of access to research and clinical services. One PPIE contributor commented: “hospitalised patients would have had some sort of intervention at some point in their illness, in stark contrast to those who were unable to access healthcare, particularly in the first wave, hence research into those may not be applicable or relevant to the vast majority of non‐hospitalised patients”. This encouraged us, as researchers, to ensure that the relevance of the study extended to people with Long Covid who were not hospitalised. Hence, we have linked with studies of community‐managed patients where possible to share findings (e.g. Convalescence study: https://www.ucl.ac.uk/covid-19-longitudinal-health-wellbeing/convalescence-long-covid-study). In addition, we have collaborated with 7 other studies from the National Long Covid Research Working Group to produce an output: ‘Patient and public involvement within epidemiological studies of Long Covid in the UK’ [2] that aimed to share PPIE successes and challenges.

**3c. Assessing the accuracy and accessibility of study information and 3 d. Assessing participant burden**

Patients initially took on a traditional PPIE role in reviewing patient‐facing materials and were asked to consider participant burden for any study amendments. This included, for example, reviewing a video illustrating the saliva sample process. In response to the PPIE review, we added written subtitles to the video, along with the audio to enable those hard of hearing or deaf to understand the video. To reduce participant burden, we separated longer visits for study participants into shorter, more regular visits as required and prioritised certain outcomes (often with the primary outcome collected first), in direct response to PPIE feedback.

**3e. Contribution to meetings**

As the PHOSP‐COVID study progressed, public contributors were linked to the relevant working groups/groups of their own interest. For instance, one patient who had pre‐existing cardiac disease was keen to join the cardiovascular working group. The sheer scale of PHOSP‐COVID, with the volume of researchers and a diverse range of work being produced, was a challenge in terms of how best to communicate with all and when to link people in at the right time. We tackled this by setting up the monthly PPIE/working group meetings and invited working group scientists/data analysts to present in the early phases of their analysis. We identified potential speakers via the PHOSP‐COVID Executive meetings and the planned publication register. PPIE contributors had an equal voice in the meeting alongside academics and clinicians with allocated space on the agenda. An example of their contribution in these meetings was to understand the public messaging from data presented by the researchers/data analysts. In many instances, they created press releases to accompany academic publications. This was a direct request from PPIE members to be involved in drafting these lay‐facing documents.

### 4.4 Public Involvement, Coordination and Documentation

**4a. Project team public involvement, knowledge and role**

Researcher engagement with the process of involvement differed across the working groups. Researcher familiarity and experience with PPIE had a significant influence on willingness and ability. For groups with less experience of PPIE, the NIHR Leicester BRC team was leveraged to offer extra guidance (co‐authors RP and JB) where required. Also, all working groups were scheduled by the PHOSP‐COVID Executive team to meet online with the PPIE group to discuss data and proposed outputs at regular intervals throughout the study lifespan. This supported a consistency of delivery and maintained a team‐wide expectation of public involvement. In total, 10 meetings took place with typically 10 PPIE contributors, 2 researchers/clinicians/data scientists from the working group, the PHOSP‐COVID PPIE Lead (lead author LHW) and one of the study Principal Investigators (co‐author RE). A buddy system was offered by the PPIE lead to the patient members if they needed additional support to attend meetings. For example, the PPIE lead could provide a lay summary of scientific reports/wording and could bring the patient members into the conversation if discussions didn't naturally happen.

**4b. Public involvement record keeping**

Formal minutes from meetings were not kept, beyond saving Teams meeting recordings and chats. Informal notes were taken by the lead author (LHW) as well as anyone else who wished to during the meeting. Presentation slides were sometimes shared between the group if the presenter was happy to share. A ‘you said, we did’ approach was adopted so that the researchers could show where they had been able to incorporate PPIE feedback or not in some instances and the reasons why, from the previous meetings. This was done verbally at the start of each meeting (i.e. recapping from the last session). For example, changes were made to lay summaries of research based on direct PPIE feedback. This helped to motivate public contributors because it demonstrated their impact on the study. It also helped to motivate researchers because they could see that the PPIE was not tokenistic, and also provided a level of accountability that helped embed PPIE.

**4c. Capturing the impact of public involvement on the research**

We have captured some public contributors and researchers quotes in the section below to showcase the impact of public involvement on the research. We are using the PIRIT tool in this manuscript to evaluate the impact and to identify any gaps, where public involvement could have been included further.

**4d. Ongoing researcher and public contributor meetings**

Efforts were made to accommodate individual needs for ongoing meetings. For example, meetings were of a duration and at a time of day to suit people with Long Covid, taking into account their symptoms (e.g. fatigue) and other commitments (e.g. lunch break), with some preferring to meet 1:1 at a time to suit. All meetings were held online to enable people to join nationally and for new members to be added over time. This facilitated accessibility and reduced the time commitment, as travel time was eliminated. All new members were upskilled and introduced to the background of the PHOSP‐COVID study to bring them up to speed, before joining a meeting.

**4e. Reflecting on public involvement activity during the research**

We have used the PIRIT tool retrospectively. In the future, it would be useful to use this tool in the planning stages and throughout the study to ensure that meaningful PPIE is considered for the entire study cycle. From our PIRIT analysis, we have been able to reflect upon how we could improve our approach to public involvement. For example, as a group we recognised that some public involvement reflections/informal activities were not being captured by meeting minutes or other recording mechanisms. Therefore, we are now using a multitude of methods to capture this activity, including: meeting recordings, saving meeting chats and updating the PIRIT tool as activity occurs.

### 4.5 The Difference That Public Contributors Make to the Research Outcomes and Dissemination of Results

**5a. Contextual interpretation of data and findings**

Lay versions of scientific outputs have been co‐created with the PPIE group (e.g. newsletters for sites, plain English summaries and media press releases). Members have linked with their charities to disseminate findings widely (e.g. Long Covid Support website and social media platforms). Two public webinars have been hosted, with researchers and public contributors co‐presenting findings and hosting panel discussions.

**5b. Dissemination strategy**

We did not create a dissemination strategy per se with our PPIE group. However, publications arising from PHOSP‐COVID are continuing (40+ to date) and we now have a publication policy for all members of the PHOSP‐COVID consortium (including our public contributors). This ensures that all members have the opportunity to contribute to and be included in publications. All public contributors included in the consortium authorship list have signed a non‐disclosure agreement (NDA) to secure research findings that are under embargo. This relates to eight of our original members who continue to work directly with the PHOSP‐COVID consortium, but who can represent and canvas the views of the tens of thousands in the peer support group of the charity Long Covid Support. We anticipated that there would be challenges with asking public contributors to sign an NDA. However, we had no reluctance from members to do this and they understood that this was required for publications under embargo.

**5c. Public involvement in academic outputs**

As the study progressed, the PPIE group worked closely with the PHOSP‐COVID working groups to ensure that key messages emerging from the data were shared with patients and the public at the early stages, rather than when results/manuscripts were fully formed. This enabled public contributors to shape the dissemination and lay messaging. On reflection, we could have asked all authors to prepare a lay summary of their outputs for the website. This would've created a valuable resource over time.

**5d. Social media**

Members have linked with their charities to disseminate findings widely. For example, Long Covid Support has used their X (formerly Twitter) channel to promote study findings/publications and tag our @PHOSP_COVID account in their communications. Social media has also been used to highlight the priority setting survey and registration for our public webinars.

### 4.6 The Difference Public Contributors Make to the Implementation of Findings

**6a. Pathway to practice and 6b. Assessing research impact: patient groups and clinical sites**

Public contributors are involved with all follow‐up project funding applications. One example was the NIHR Policy research proposal, which was successfully funded and started on 01 July 2021. This project evaluated the clinical and cost effectiveness of Long Covid services in the UK [16]. This evaluated the individual sites from PHOSP‐COVID in terms of availability and specifications of Long Covid clinics, rehabilitation and mental health services. A project advisory group (PAG) was established for this study, which included members from the existing PHOSP‐COVID PPIE group. For example, we included non‐hospitalised patients as eligible for the PHOSP‐I study, based on feedback from the PPIE group.

The inclusion of the patient voice in our end‐of‐study summit meeting and at other times was also hugely motivating for researchers. For example, our patient contributors created testimonial videos of their Long Covid and PPIE experiences, which were played to clinicians and researchers at events such as the summit meeting, public webinars and Medical Research Council Awards, to showcase research impact.

Public contributors were involved in the consideration of how the findings from the research would be embedded in clinical practice. For example, findings from the NIHR Policy research proposal (described above) were considered by the PAG and as a result, shared with charities such as Long Covid Support to feed into their policy reports: https://www.longcovid.org/research/our-research and the UK Covid‐19 inquiry.

### 4.7 Public Contributor Debriefing

**7a. Assessing the outcomes of planned public involvement activity and how public involvement has informed the project**

**Figure 4:**
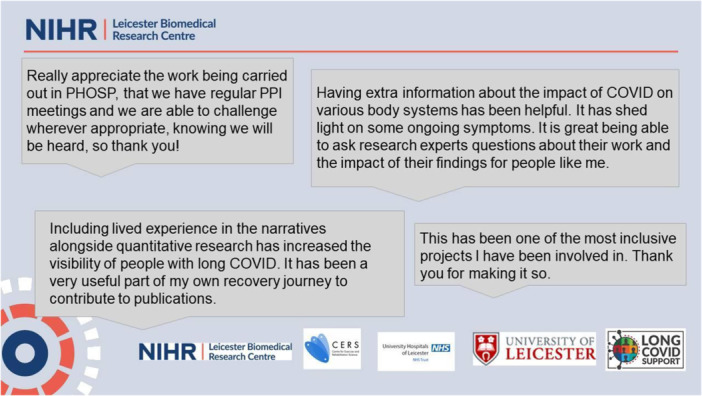
Quotes from public contributors, from the annual survey‐ December 2024.

It was important to researchers and the patient members to provide feedback to patients during the course of the work to demonstrate the impact of their involvement. We did this via ‘you said, we did’ sessions and proactively sought (and acted upon) feedback from patients about the PPIE process itself at appropriate times. For example, when considering how best to include participants early in work for publication, patient representatives called for meetings with the working groups to present their data at an early stage before firm conclusions had been made. We also conduct an anonymous annual survey to canvass the views of the public contributors on their involvement in the last year and to check if/how they'd like to continue contributing (Figure 4).

### 4.8 Reporting the Difference Public Involvement Has Made to the Research and Lessons Learned

**8a. Reporting and promoting public involvement impact**

We seek to share the good practice we have developed in PHOSP‐COVIDPPIE, with this publication as an example. We may also include networking with regional and national PPIE strategy teams (e.g. NIHR) to amplify sharing of learnings with other PPIE colleagues.

**8b. Public involvement learning and development**

Public contributors have shared their public involvement learning and good practice with a wide audience via our public webinars, publications [17] and testimonial videos. This has been in relation to informal training they have received in the lifecourse of the project rather than formal training programmes.

### 4.9 Reflections from Public Contributors and Researchers

It is important to reflect upon the challenges of consortium‐driven PPIE as well as the benefits.

***Feedback from one public contributor:** PHOSPbrought together a team of expert researchers and clinicians, and injected into the mix those of us with lived experience of Long Covid. This resulted in a consortium with the knowledge and skills to approach Long Covid research. Having the patient voice at the heart of research really helps ensure that the condition is understood, the research remains relevant and that effective results are achieved. Those people working in PPI groups are not only patients; they may have a lot of other skills too that can really benefit a study. One of the main tasks PHOSP asked us to support with, was to generate a list of priority areas for research. So we designed and publicised a survey to get the views of a large number of people. The findings were used at a study‐wide event where patients were included as equals. That resulted in a list of questions that steered subsequent research. But not only did PHOSP concentrate on the top 10 questions, all of the questions were made publicly available so that anyone could use them to advance the research into Covid‐19. Thank you to all of you who are conducting quality research with proper patient involvement. And please don't stop, because there are millions of people worldwide for whom this research must deliver*.

***Feedback from one researcher:** Here is how PPIE has influenced how we wrote our paper. We had identified that two biomarker profiles predicted cognitive deficits in the months after COVID hospitalisation. One biomarker profile was also associated with signs of occupational impact, whereas the other was not. Initially, we had claimed that one biomarker profile was not associated with occupational impact. After discussion within the PPIE, we understood that this could be grossly misrepresented as “brain fog without consequences”. We rephrased the manuscript to make sure that we focussed on positive findings (i.e. the presence of occupational impact for one profile) and that whenever we referred to the negative finding, we made it clear that it is the absence of “evidence of occupational impact” as measured by occupational changes and difficulty working. In other words, we used wording that made it clear that the absence of evidence is not evidence of absence*.

***Feedback from one PPIE contributor:** Being an unknown virus and knowing that I'd survived when others had not, I didn't hesitate to say yes to signing up to the trial. I have since gone on to join the PPIE group following the study. If I can help others by doing my bit to support the cause, i.e vaccines, research etc, it could have global implications, so I thought why not. The benefits for me have been that I have seen my own progress through the trial, the research visits gave me peace of mind. I have enjoyed being part of the PPIE group, knowing that I may be helping patients in the future*.

### 4.10 Top Tips for Researchers to Conduct Excellent Ppie in the Future

We have linked our top tips for researchers back to the *Going the Extra Mile* document [8] and the NIHR Standards and reflected on gaps in the PIRIT tool that we can take forward to future work (Table 1).

**Table 1:** PPIE top tips for researchers, linked to NIHR standards.

Top tip	Links to….
Consider the time, location, frequency and duration of meetings/PPIE activities. What can you put in place to support members to be more involved? For example, online meetings may increase the reach.	Reach – developing practices that are more inclusive and involve people representative of the target populations.
Show the PPIE group the impact they have had on your research with a ‘you said, we did’ approach for feeding back.	Refinement and improvement –improving practice to avoid tokenism. Impact ‐ Seek improvement by identifying and sharing the difference that public involvement makes to research.
Take time to establish a PPIE group that is representative of your population/condition/local context. For Long Covid this involved engaging with a number of patient charities and our local communities.	Relevance – ensuring research is relevant to and benefits local communities.
Meet with the groups regularly (e.g. monthly), even if you don't have much to update on. Check in with them and build relationships. Provide a space for people in the group to socialise with each other, without an agenda.	Relationships – building effective relationships with local communities and individuals.
Recognise public contributors as experts in their own right and view them holistically. Value their lived experiences, and consider the skills they have in addition to being an expert patient. Give public contributors an equal opportunity to speak as per clinicians and researchers. This may include careful chairing of meetings with scheduled space on the agenda for public contributors to contribute. This should not just be at the end of the agenda, to avoid being perceived as an afterthought.	Working Together‐ Work together in a way that values all contributions and that builds and sustains mutually respectful and productive relationships.
Work with charity groups, e.g. Long Covid Support, to widen reach. Consider accessibility of meetings in terms of venue and time, considering that, although online meetings may maximise reach, they will exclude those without digital literacy/resources.	Inclusive Opportunities‐ Offer public involvement opportunities that are accessible and that reach people and groups according to research needs.
Deliver relevant training for public contributors. e.g. briefing on the research study, how to review documents, writing a lay summary etc. Signpost to other relevant training opportunities to build confidence e.g. NIHR Learn. Offer a buddy system between a PPIE lead in the research team and public contributor for extra support where required.	Support and Learning: Offer and promote support and learning opportunities that build confidence and skills for public involvement in research.
Embed PPIE into the study governance structure from the start (e.g. within the Executive or Trial Steering Group) to ensure accountability for management, regulation, leadership, resourcing and decision making. Create terms of reference so that everyone is aware of their roles and responsibilities.	Governance‐ Involve the public in research management, regulation, leadership and decision making.
Co‐produce patient/public‐facing documents and outputs with public contributors e.g. public newsletters, webinars, patient materials and plain English press releases. Circulate agenda and meeting papers at least one week ahead of the meeting to allow time for public contributors to review these. Fund the time taken to review these documents.	Communication‐ Use plain language for well‐timed and relevant communications, as part of involvement plans and activities.
Measure the impact of PPIE on your research using an evidence‐based tool e.g. PIRIT. Use this tool from the start of your research.	Impact‐ Seek improvement by identifying and sharing the difference that public involvement makes to research.

## 5 Discussion

In the PHOSP‐COVID consortium, we have embedded PPIE within the project throughout the research cycle from research prioritisation and identification of new research topics to dissemination, including co‐authored publications. This approach is an alternative method whereby patient and public involvement has moved beyond project‐specific activities and is embedded within the wider infrastructure and governance of the consortium. Barriers or challenges to traditional study PPIE beyond tokenism are well documented [5]. There is limited information on managing PPIE within a consortium, and we have encountered specific challenges with the PHOSP‐COVID study. There is some evidence that there are advantages to approaching public involvement at a consortium or organisational level, including development of more meaningful and sustained relationships with public contributors and community organisations, a broader understanding of the research agenda in the context of organisational strategic considerations and cost‐efficiency. This underpins a recommendation to embed project‐specific public involvement into models of involvement that establish public contributors and community relationships as part of the whole system of the organisation or consortium, including in both governance and operational functions. However, it requires adequate resourcing [18] and cultural change. These benefits and challenges have been outlined in this manuscript.

There are a number of tools available to measure PPIE impact; however, the impact assessment is not always done in the best way to give the communities involved what they need. Impact is contingent on the quality of public involvement, which in turn demands a rigorous examination of the effectiveness of public involvement approaches and strategies. Challenges that are already recognised include a narrowness of existing models of involvement, likely related to a neglect of equality, diversity and inclusion considerations leading to ethnocentric approaches, and a failure to assess impact consistently (or at all) [1]. Other challenges include limited awareness among communities of opportunities for involvement, inconsistent resourcing, varied and inconsistent policy and practice between organisations, patchy training and support [1]. The PIRIT tool [12]was chosen in this manuscript to evaluate PPIE impact within the PHOSP‐COVID study. Our positive findings broadly map to relevant UK public involvement standards [8, 9].

### 5.1 Legacy and Future Work

#### 5.1.1 Study Legacy

The main cohort study follow‐up finished in March 2023, but the spin‐off studies continue, including PHOSP‐I: a clinical trial of Tocilizumab to investigate the effect on health‐related quality of life in adults with Long COVID and persistent inflammation. Members of the original PHOSP PPIE group continue to support this trial. For example, PPIE contributors have created patient recruitment videos that have been shared online and developed a pre‐visit checklist for recruiting sites to complete with patients prior to their visit. The aim of the checklist is to reduce patient burden for the face‐to‐face visits. Furthermore, as a result of feedback from the PPIE group, the PHOSP‐I study was designed to include people with Long Covid who were not hospitalised with their acute Covid infection.

This manuscript has identified gaps in PPIE considerations for the PHOSP‐COVID study and specific challenges around a consortium‐based approach for PPIE that we would address in future work. These are largely due to time scale (i.e. the pace of setting up the study within a pandemic) and communication factors (diverse and large numbers of people to include/inform). These challenges have been echoed by others engaged in Long Covid research [19]. Through reflection on the challenges and successes experienced in the PHOSP_COVID project PPIE, we have developed recommendations to support future good practice.

There remains a limited understanding of why some people develop Long Covid, its mechanisms and potentially effective interventions; therefore, continuing to have patients at the heart of future research in this area remains essential.

## 6 Conclusion

This manuscript has identified PPIE considerations and challenges around a consortium‐based approach for PPIE from the PHOSP‐COVID study. These are largely due to time scale (i.e. the pace of setting up the study within a pandemic) and communication factors (diverse and large numbers of people to include/inform). Through reflection on the challenges and successes experienced in the PHOSP‐COVID consortium PPIE via a PIRIT‐focused impact evaluation, we have developed recommendations to support future good practice.

## Author Contributions

L.H.W. and R.E. agree to be accountable for all aspects of the work, ensuring that any questions related to the accuracy or integrity of any part of the work are appropriately investigated and resolved. L.H.W., R.E., N.S., C.H., N.R., R.G., R.N., J.B., L.S. and D.A. made substantial contribution to the conception or design of the work. L.H.W., R.E., J.B. and R.B. drafted the work. All authors critically reviewed the work for important intellectual content and read and approved the final manuscript.

## Ethics Statement

The main PHOSP‐COVID study ethics approval was granted by Leeds West Research Ethics Committee, Reference 20/YH/0225.

## Conflicts of Interest

The authors declare that they have no competing interests in relation to this manuscript.

## Data Availability

Data sharing not applicable to this article as no datasets were generated or analysed during the current study.
